# Supplementary material for: Morphological and Molecular Characterization of Three Endolichenic Isolates of Xylaria (Xylariaceae), from Cladonia curta Ahti & Marcelli (Cladoniaceae)
Source: Plants (Basel). 2019 Oct 8;8(10):399. doi: 10.3390/plants8100399 (PMC6843379; doi:10.3390/plants8100399)
Supplement: Supplementary file 1 [file plants-08-00399-s001.pdf]

Supplementary Material

Table S1. Genbank accession numbers and references for ITS region of rDNA sequences of taxa included in phylogenetic analyses in this study.

Accession	Reference	Accession	Reference
AY315386	Davis et al., 2003	KT289545	Arnold et al., 2009
AY315387	Davis et al., 2003	KT289546	Arnold et al., 2009
AY315388	Davis et al., 2003	KT289547	Arnold et al., 2009
AY315389	Davis et al., 2003	KT289548	Arnold et al., 2009
AY315390	Davis et al., 2003	KT289549	Arnold et al., 2009
AY315391	Davis et al., 2003	KT289550	Arnold et al., 2009
AY315393	Davis et al., 2003	KT289551	Arnold et al., 2009
AY315394	Davis et al., 2003	KT289552	Arnold et al., 2009
AY315395	Davis et al., 2003	KT289553	Arnold et al., 2009
AY315396	Davis et al., 2003	KT289554	Arnold et al., 2009
AY315400	Davis et al., 2003	KT289555	Arnold et al., 2009
AY315401	Davis et al., 2003	KT289556	Arnold et al., 2009
AY315402	Davis et al., 2003	KT289557	Arnold et al., 2009
AY315404	Davis et al., 2003	KT289558	Arnold et al., 2009
AY315405	Davis et al., 2003	KT289559	Arnold et al., 2009
AY315406	Davis et al., 2003	KT289560	Arnold et al., 2009
AY315407	Davis et al., 2003	KT289561	Arnold et al., 2009
KT289521	Arnold et al., 2009	KT289562	Arnold et al., 2009
KT289522	Arnold et al., 2009	KT289563	Arnold et al., 2009
KT289523	Arnold et al., 2009	KT289564	Arnold et al., 2009
KT289524	Arnold et al., 2009	KT289565	Arnold et al., 2009
KT289525	Arnold et al., 2009	KT289566	Arnold et al., 2009
KT289526	Arnold et al., 2009	KT289567	Arnold et al., 2009
KT289527	Arnold et al., 2009	KT289568	Arnold et al., 2009
KT289528	Arnold et al., 2009	KT289569	Arnold et al., 2009
KT289529	Arnold et al., 2009	KT289570	Arnold et al., 2009
KT289530	Arnold et al., 2009	KT289571	Arnold et al., 2009
KT289531	Arnold et al., 2009	KT289572	Arnold et al., 2009
KT289532	Arnold et al., 2009	KT289573	Arnold et al., 2009
KT289533	Arnold et al., 2009	KT289574	Arnold et al., 2009
KT289534	Arnold et al., 2009	KT289575	Arnold et al., 2009
KT289535	Arnold et al., 2009	KT289576	Arnold et al., 2009
KT289536	Arnold et al., 2009	KT289577	Arnold et al., 2009
KT289537	Arnold et al., 2009	KT289578	Arnold et al., 2009
KT289538	Arnold et al., 2009	KT289579	Arnold et al., 2009
KT289539	Arnold et al., 2009	KT289580	Arnold et al., 2009
KT289540	Arnold et al., 2009	KT289581	Arnold et al., 2009
KT289541	Arnold et al., 2009	KT289582	Arnold et al., 2009
KT289542	Arnold et al., 2009	KT289583	Arnold et al., 2009
KT289543	Arnold et al., 2009	KT289584	Arnold et al., 2009
KT289544	Arnold et al., 2009	KT289585	Arnold et al., 2009
KT289586	Arnold et al., 2009	KT289634	Arnold et al., 2009
KT289587	Arnold et al., 2009	KT289635	Arnold et al., 2009
KT289588	Arnold et al., 2009	JQ759362	U´Ren et al., 2016
KT289589	Arnold et al., 2009	JQ759383	U´Ren et al., 2016
KT289590	Arnold et al., 2009	JQ760192	U´Ren et al., 2016
KT289591	Arnold et al., 2009	JQ760209	U´Ren et al., 2016

KT289592	Arnold et al., 2009	JQ760257	U´Ren et al., 2016
KT289593	Arnold et al., 2009	JQ760320	U´Ren et al., 2016
KT289594	Arnold et al., 2009	JQ760565	U´Ren et al., 2016
KT289595	Arnold et al., 2009	JQ760593	U´Ren et al., 2016
KT289596	Arnold et al., 2009	JQ760665	U´Ren et al., 2016
KT289597	Arnold et al., 2009	JQ760666	U´Ren et al., 2016
KT289598	Arnold et al., 2009	JQ761035	U´Ren et al., 2016
KT289599	Arnold et al., 2009	JQ761586	U´Ren et al., 2016
KT289600	Arnold et al., 2009	JQ761635	U´Ren et al., 2016
KT289601	Arnold et al., 2009	JQ761899	U´Ren et al., 2016
KT289602	Arnold et al., 2009	JQ761992	U´Ren et al., 2016
KT289603	Arnold et al., 2009	HM122805	U´Ren et al., 2016
KT289604	Arnold et al., 2009	HM123248	U´Ren et al., 2016
KT289605	Arnold et al., 2009	HM123416	U´Ren et al., 2016
KT289606	Arnold et al., 2009	JQ760181	U´Ren et al., 2016
KT289607	Arnold et al., 2009	JQ760182	U´Ren et al., 2016
KT289608	Arnold et al., 2009	JQ760210	U´Ren et al., 2016
KT289609	Arnold et al., 2009	JQ760306	U´Ren et al., 2016
KT289610	Arnold et al., 2009	JQ760314	U´Ren et al., 2016
KT289611	Arnold et al., 2009	JQ760457	U´Ren et al., 2016
KT289612	Arnold et al., 2009	JQ760469	U´Ren et al., 2016
KT289613	Arnold et al., 2009	JQ760489	U´Ren et al., 2016
KT289614	Arnold et al., 2009	JQ760548	U´Ren et al., 2016
KT289615	Arnold et al., 2009	JQ760549	U´Ren et al., 2016
KT289616	Arnold et al., 2009	JQ760650	U´Ren et al., 2016
KT289617	Arnold et al., 2009	JQ760654	U´Ren et al., 2016
KT289618	Arnold et al., 2009	JQ760728	U´Ren et al., 2016
KT289619	Arnold et al., 2009	JQ760786	U´Ren et al., 2016
KT289620	Arnold et al., 2009	JQ760795	U´Ren et al., 2016
KT289621	Arnold et al., 2009	JQ760869	U´Ren et al., 2016
KT289622	Arnold et al., 2009	JQ760904	U´Ren et al., 2016
KT289623	Arnold et al., 2009	JQ760970	U´Ren et al., 2016
KT289624	Arnold et al., 2009	JQ760995	U´Ren et al., 2016
KT289625	Arnold et al., 2009	JQ761025	U´Ren et al., 2016
KT289626	Arnold et al., 2009	KT289630	Arnold et al., 2009
KT289627	Arnold et al., 2009	KT289631	Arnold et al., 2009
KT289628	Arnold et al., 2009	KT289632	Arnold et al., 2009
KT289629	Arnold et al., 2009	KT289633	Arnold et al., 2009

Table S2. Genbank accession numbers and references for β-Tubulin sequences of taxa included in phylogenetic analyses in this study.

Accession	Reference
GQ502698	Hsieh et al., 2010
KU684111	U´Ren et al., 2016
KU684112	U´Ren et al., 2016
KU684121	U´Ren et al., 2016
KU684122	U´Ren et al., 2016
KU684141	U´Ren et al., 2016
KU684142	U´Ren et al., 2016
KU684143	U´Ren et al., 2016
KU684144	U´Ren et al., 2016

KU684145	U´Ren et al., 2016
KU684146	U´Ren et al., 2016
KU684147	U´Ren et al., 2016
KU684148	U´Ren et al., 2016
KU684153	U´Ren et al., 2016
KU684154	U´Ren et al., 2016
KU684156	U´Ren et al., 2016
KU684157	U´Ren et al., 2016
KU684158	U´Ren et al., 2016
KU684159	U´Ren et al., 2016
KU684164	U´Ren et al., 2016
KU684165	U´Ren et al., 2016
KU684167	U´Ren et al., 2016
KU684168	U´Ren et al., 2016
KU684170	U´Ren et al., 2016
KU684171	U´Ren et al., 2016
KU684173	U´Ren et al., 2016
KU684174	U´Ren et al., 2016
KU684175	U´Ren et al., 2016
KU684176	U´Ren et al., 2016
KU684177	U´Ren et al., 2016
KU684178	U´Ren et al., 2016
KU684179	U´Ren et al., 2016
KU684193	U´Ren et al., 2016
KU684197	U´Ren et al., 2016
KU684198	U´Ren et al., 2016
KU684201	U´Ren et al., 2016

Table S3. Nucleotide substitution models of group of sequences established in order to provi de a phylogenetic context for the isolated endolichenic fungi from *Cladonia curta* based in ITS region of rDNA. The groups of sequences (G) were: (1) BLASTn search; (2) Arnold et al., 2009; (3) U'Ren et al., 2016 and (4) David et al., 2003. BIC: Bayesian Information Criterion; AICc: Akaike Information Criterion, corrected; *lnL*: Maximum Likelihood value; P: number of parameters including branch lengths. *R*: Values of transition/transversion bias; *f*: nucleotide frequencies; *r*: rates of base substitutions; +*G*: Gamma distribution; +*I*: Invariant sites; HKY: Hasegawa-Kishino-Yano; TN93: Tamura-Nei; T92: Tamura 3-parameter; K2: Kimura 2-parameter.

G	Model	P	BIC	AICc	*lnL*	(+*I*)	(+*G*)	*R*	*f*(A)	*f*(T)	*f*(C)	*f*(G)	*r*(AT)	*r*(AC)	*r*(AG)	*r*(TA)	*r*(TC)	*r*(TG)	*r*(CA)	*r*(CT)	*r*(CG)	*r*(GA)	*r*(GT)	*r*(GC)
1	K2+G+I	58	5708.971	5271.012	−2577.263	0.25	1.21	1.56	0.250	0.250	0.250	0.250	0.049	0.049	0.152	0.049	0.152	0.049	0.049	0.152	0.049	0.152	0.049	0.049
	K2+I	57	5709.381	5278.965	−2582.248	0.38	n/a	1.47	0.250	0.250	0.250	0.250	0.051	0.051	0.149	0.051	0.149	0.051	0.051	0.149	0.051	0.149	0.051	0.051
	K2+G	57	5723.389	5292.973	−2589.252	n/a	1.21	1.34	0.250	0.250	0.250	0.250	0.053	0.053	0.143	0.053	0.143	0.053	0.053	0.143	0.053	0.143	0.053	0.053
	T92+G+I	59	5723.673	5278.171	−2579.835	0.25	1.18	1.55	0.256	0.256	0.244	0.244	0.050	0.048	0.148	0.050	0.148	0.048	0.050	0.156	0.048	0.156	0.050	0.048
2	TN93+G+I	242	13900.845	11895.883	−5703.950	0.33	0.50	1.79	0.242	0.256	0.248	0.254	0.046	0.044	0.112	0.043	0.208	0.046	0.043	0.215	0.046	0.106	0.046	0.044
	K2+G+I	238	13903.158	11931.270	−5725.709	0.34	0.52	1.80	0.250	0.250	0.250	0.250	0.045	0.045	0.161	0.045	0.161	0.045	0.045	0.161	0.045	0.161	0.045	0.045
	T92+G+I	239	13911.795	11931.639	−5724.877	0.34	0.52	1.80	0.249	0.249	0.251	0.251	0.044	0.045	0.161	0.044	0.161	0.045	0.044	0.160	0.045	0.160	0.044	0.045
	HKY+G+I	241	13926.847	11930.153	−5722.102	0.34	0.52	1.80	0.242	0.256	0.248	0.254	0.046	0.044	0.163	0.043	0.159	0.045	0.043	0.165	0.045	0.156	0.046	0.044
3	K2+G	93	9811.489	9103.878	−4458.356	n/a	0.26	2.09	0.250	0.250	0.250	0.250	0.040	0.040	0.169	0.040	0.169	0.040	0.040	0.169	0.040	0.169	0.040	0.040
	K2+G+I	94	9814.955	9099.748	−4455.278	0.37	0.74	2.11	0.250	0.250	0.250	0.250	0.040	0.040	0.170	0.040	0.170	0.040	0.040	0.170	0.040	0.170	0.040	0.040
	T92+G	94	9823.774	9108.568	−4459.688	n/a	0.26	2.09	0.259	0.259	0.241	0.241	0.042	0.039	0.163	0.042	0.163	0.039	0.042	0.175	0.039	0.175	0.042	0.039
	T92+G+I	95	9826.986	9104.183	−4456.483	0.37	0.73	2.12	0.259	0.259	0.241	0.241	0.041	0.039	0.164	0.041	0.164	0.039	0.041	0.176	0.039	0.176	0.041	0.039
4	K2+G+I	56	5722.084	5323.500	−2605.402	0.39	0.87	2.00	0.250	0.250	0.250	0.250	0.042	0.042	0.167	0.042	0.167	0.042	0.042	0.167	0.042	0.167	0.042	0.042
	K2+G	55	5722.243	5330.764	−2610.046	n/a	0.29	2.02	0.250	0.250	0.250	0.250	0.041	0.041	0.167	0.041	0.167	0.041	0.041	0.167	0.041	0.167	0.041	0.041
	T92+G	56	5723.685	5325.102	−2606.203	n/a	0.29	2.05	0.268	0.268	0.232	0.232	0.044	0.038	0.156	0.044	0.156	0.038	0.044	0.181	0.038	0.181	0.044	0.038
	T92+G+I	57	5724.678	5318.989	−2602.134	0.39	0.82	2.04	0.268	0.268	0.232	0.232	0.044	0.038	0.156	0.044	0.156	0.038	0.044	0.180	0.038	0.180	0.044	0.038

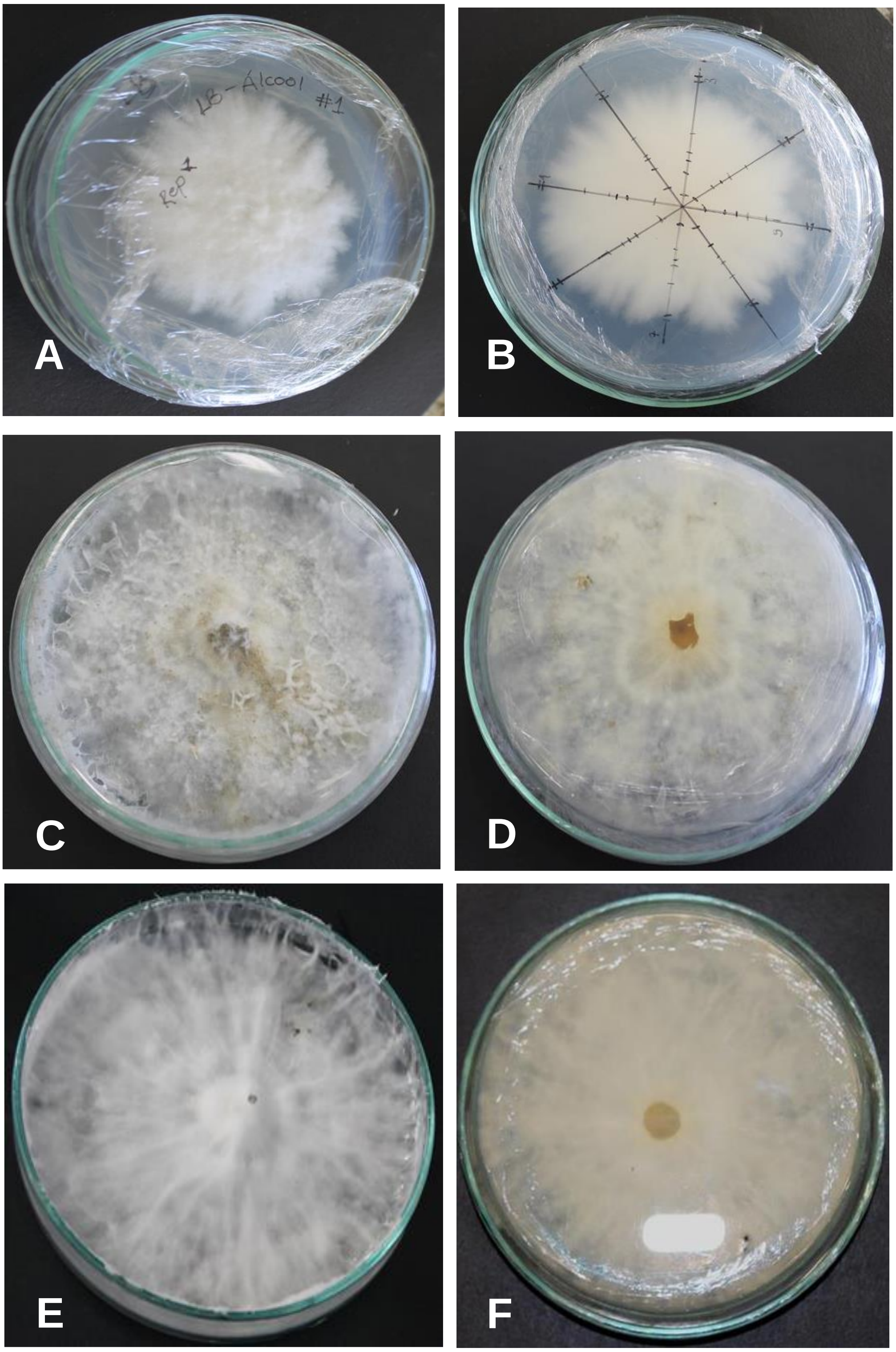

Figure S1. Appearance of isolated *Xylaria* sp. 1 (HBEI 001), colonies on MS medium. **A, B**. Pure culture of the isolated fungi. **C, D**. Appearance of endolichenic fungi in photoperiod of 16 h light / 8 h dark. **E, F**. Appearance of endolichenic fungi in darkness.

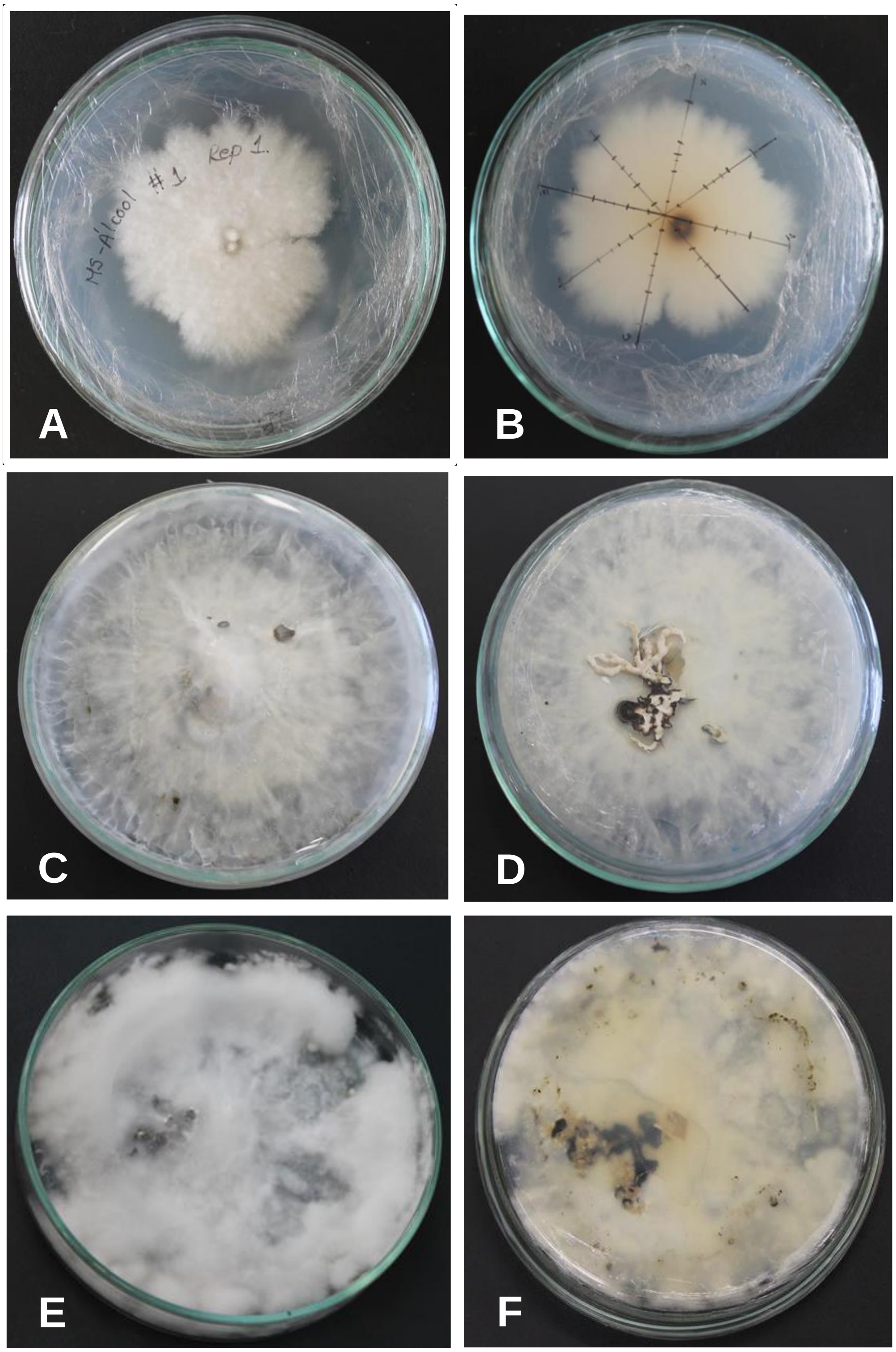

Figure S2. Appearance of isolated *Xylaria* sp. 2 (HBEI 002), colonies on MS medium. **A, B**. Pure culture of the isolated fungi. **C, D**. Appearance of endolichenic fungi in photoperiod of 16 h light / 8 h dark. **E, F**. Appearance of endolichenic fungi in darkness.

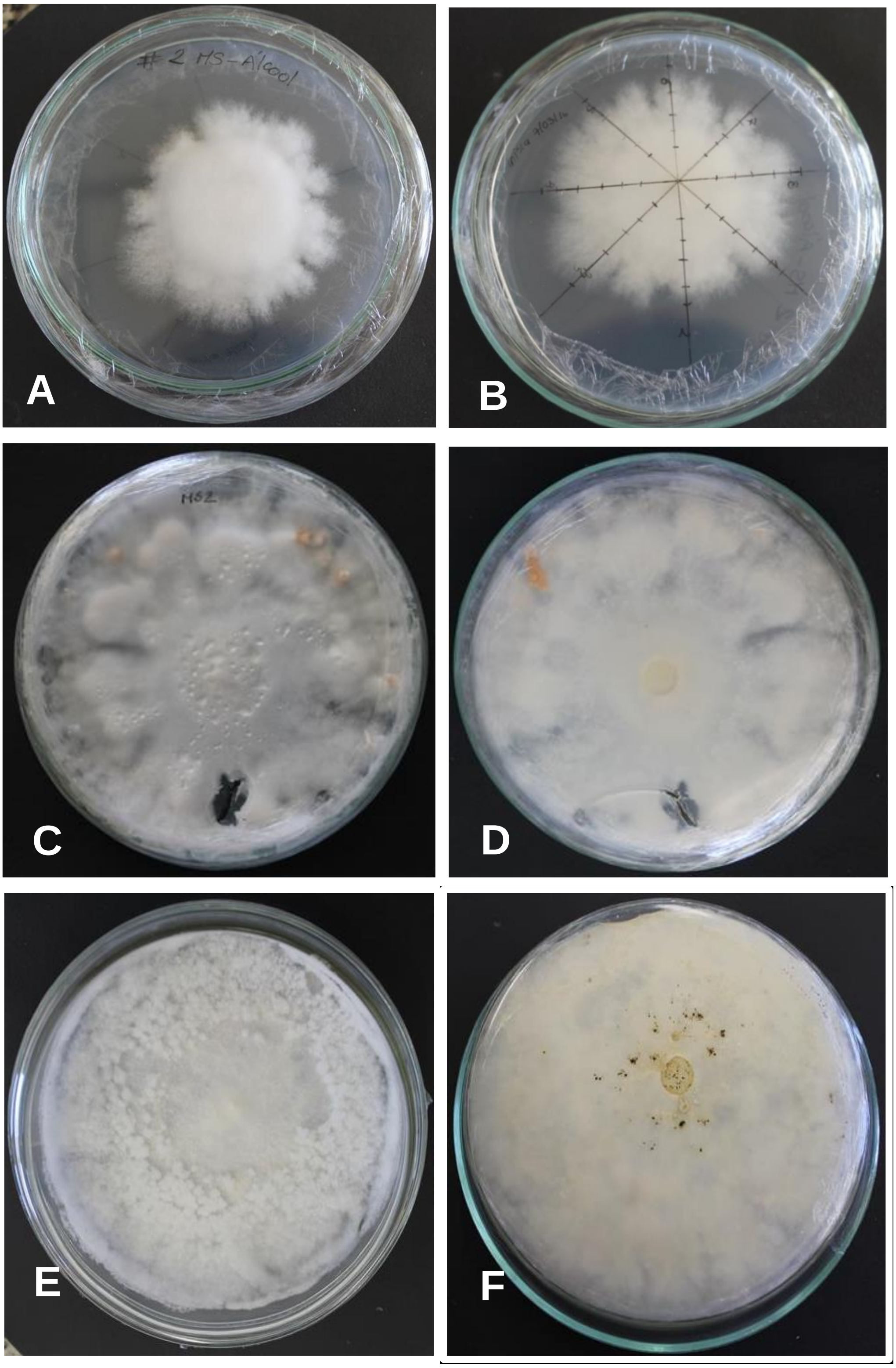

Figure S3. Appearance of isolated *Xylaria* sp. 3 (HBEI 003), colonies on MS medium. **A, B**. Pure culture of the isolated fungi. **C, D**. Appearance of endolichenic fungi in photoperiod of 16 h light / 8 h dark. **E, F**. Appearance of endolichenic fungi in darkness.
